# Intervertebral Disc Degeneration: Biomaterials and Tissue Engineering Strategies toward Precision Medicine

**DOI:** 10.1002/adhm.202102530

**Published:** 2022-05-04

**Authors:** Isma Liza Mohd Isa, Sabarul Afian Mokhtar, Sunny A. Abbah, Mh Busra Fauzi, Aiden Devitt, Abhay Pandit

**Affiliations:** ^1^ Department of Anatomy Faculty of Medicine Universiti Kebangsaan Malaysia Kuala Lumpur 56000 Malaysia; ^2^ CÚRAM SFI Research Centre for Medical Devices National University of Ireland Galway H91W2TY Ireland; ^3^ Department of Orthopaedics and Traumatology Faculty of Medicine Universiti Kebangsaan Malaysia Kuala Lumpur 56000 Malaysia; ^4^ Centre for Tissue Engineering and Regenerative Medicine Faculty of Medicine Universiti Kebangsaan Malaysia Kuala Lumpur 56000 Malaysia; ^5^ Department of Orthopedic Surgery University Hospital Galway Galway H91YR71 Ireland

**Keywords:** biomaterials, discogenic low back pain, intervertebral discs, precision medicine, tissue engineering

## Abstract

Intervertebral disc degeneration is a common cause of discogenic low back pain resulting in significant disability. Current conservative or surgical intervention treatments do not reverse the underlying disc degeneration or regenerate the disc. Biomaterial‐based tissue engineering strategies exhibit the potential to regenerate the disc due to their capacity to modulate local tissue responses, maintain the disc phenotype, attain biochemical homeostasis, promote anatomical tissue repair, and provide functional mechanical support. Despite preliminary positive results in preclinical models, these approaches have limited success in clinical trials as they fail to address discogenic pain. This review gives insights into the understanding of intervertebral disc pathology, the emerging concept of precision medicine, and the rationale of personalized biomaterial‐based tissue engineering tailored to the severity of the disease targeting early, mild, or severe degeneration, thereby enhancing the efficacy of the treatment for disc regeneration and ultimately to alleviate discogenic pain. Further research is required to assess the relationship between disc degeneration and lower back pain for developing future clinically relevant therapeutic interventions targeted towards the subgroup of degenerative disc disease patients.

## Graphical abstract

Understanding the patient‐specific disease mechanisms underlying the severity of disc degeneration is essential for identifying therapeutic targets to design biomaterials and tissue engineering toward precision therapy. Targeted biomaterials at an early stage or the combination of biomaterials and therapeutic molecules or cells via the minimally invasive injection or surgical approach at an advanced stage can improve the treatment efficacy.

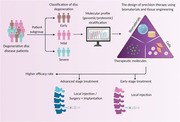

## 1 Introduction

Low back pain (LBP) is a common health problem, causing significant life years of disability as reported by the Global Burden of Disease Study.^[^ ^1^ ^]^ A prevalence study indicated that the lower back was the most common site of pain, accounting for 47.6% of chronic pain cases in Ireland.^[^ ^2^ ^]^ Approximately €28 million was spent on spinal fusions and spine‐related procedures in hospitals for chronic LBP in Ireland.^[^ ^3^ ^]^ In comparison, the annual healthcare costs of LBP‐related issues were reported at £19.77 billion in the United Kingdom^[^ ^4^ ^]^ and $100 billion in the United States.^[^ ^5^ ^]^

The etiology of LBP is not fully understood and multifactorial but degeneration of the intervertebral disc (IVD) undoubtedly plays a part. Pain may result directly from damage to the disc and inflammatory changes in the adjacent endplates or indirectly from changes in the sagittal balance of the spine caused by disc height reduction leading to muscle fatigue pain. The prevalence study estimated that between 26% and 42% of degenerative disc disease patients experience low back pain.^[^ ^6^ ^]^ The IVD is a fibrocartilaginous tissue connecting adjacent vertebrae. It provides mechanical stability to the spine by transmitting the load and allows flexion, extension, side bending, and rotation at the motion segment level. The IVD comprises a central core proteoglycan‐rich gelatinous nucleus pulposus (NP) enclosed peripherally by the lamellae of collagenous annulus fibrosus (AF). The endplate of IVD is composed of cartilaginous tissue that superiorly and inferiorly connects the adjacent vertebral bodies forming the functional spinal unit.^[^ ^7^ ^]^ The IVD degeneration is associated with dysregulation of extracellular matrix (ECM) homeostasis that is caused by a decrease of ECM synthesis and an increase in ECM degradation, leading to inflammation. The onset of discogenic LBP has been correlated with the hyperinnervation of sensory nerve fibers into the aneural disc, which initiates nociception. The inflammatory mediators and neurotrophins induce nerve ingrowth, sensory sensitization in the degenerative disc, resulting in discogenic LBP.^[^ ^8^ ^]^ Current treatment includes medication, rehabilitation, and spinal surgeries, aiming to alleviate pain, nevertheless do not address the pathology underlying IVD degeneration.

This review focuses on the pathogenesis of IVD degeneration associated discogenic pain and current clinical treatment. We also highlight the emerging concept of precision medicine in IVD tissue engineering, in particular the personalize biomaterial‐based tissue engineering tailored to the severity of the disc degeneration at early, mild or advanced stages, the ongoing and concluded clinical trials and future directions.

## 2 Intervertebral Disc Degeneration

**Figure 1:**
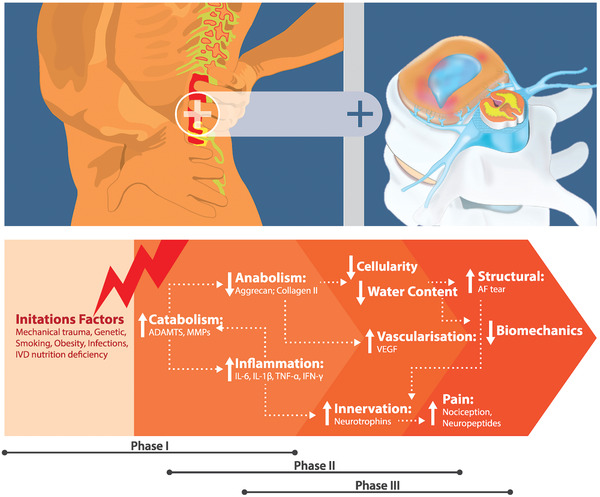
Schematic summarizes the phases of intervertebral disc degeneration associated discogenic pain. A multifactorial condition initiates an imbalance of ECM metabolism in the early phase of degeneration by increasing degradative enzymes such as ADAMTS and MMPs to promote ECM degradation and reduce ECM synthesis, including aggrecan and collagen. Alteration of ECM biochemical compositions induce the production of proinflammatory mediators includes IL‐1*β* TNF, IFN and IL‐6. In phase II, inflammatory insult results in further ECM degradation and loss of cell density. Further, proinflammatory cytokines stimulate AF and NP cells and infiltration of immune cells to release growth factors such as VEGF and neurotrophins such as NGF and BDNF to promote neovascularization and nerve in‐growth in the IVD. Phase III is characterized by continuous neurogenic insults to induce pronociceptive molecules such as neuropeptides to be released in IVD and spinal level, and activation of pronociceptor to sensitize and develop pain. Advanced structural breakdown results in annular tears and mechanical instability, contributing to disc herniation.

The onset of IVD degeneration begins in young adults and progresses with age and ongoing degeneration under pathological insults. The pathophysiology of IVD degeneration is described as Phase I, Phase II and Phase III for early, mild and severe stages (**Figure** **1**).

### 2.1 Phase I—Early Degeneration

The early stage of disc degeneration is associated with the biochemical content of the ECM changes in the NP as type II collagen, proteoglycan (PG), and water content decreases, resulting in reduced axial loads in the AF. An increase in cellular apoptosis during ageing can cause low cellularity and phenotypic changes in cells, all of which result in a loss of the cell's ability to synthesize ECM content, causing dysregulation of ECM homeostasis.^[^ ^9^ ^]^ Enzymes mediating ECM degradation, including matrix metalloproteinases (MMPs) and aggrecanases, also increase during degeneration and ageing. The most significant dysregulation of biochemical content due to ECM degradation is the loss of proteoglycan that initiates the degradation of aggrecan, shifting the glycosaminoglycan (GAG) proportion from chondroitin sulfate to keratan sulfate, and increasing other ECM compositions such as fibronectin, versican, biglycan, and decorin.^[^ ^10^ ^]^ The lamellae of the AF become disorganized as degeneration progresses, with type I collagen generating stronger collagen fibrils predominately found in the NP and inner AF. The osmotic pressure declines with the loss of the water content, making the disc less hydrated and resulting in disc collapse. The prolonged depletion of proteoglycan causes the movement of large uncharged molecules such as serum proteins, proinflammatory cytokines, and neurogenic mediators into the disc that can induce inflammation and progression of disc degeneration.^[^ ^11^ ^]^

### 2.2 Phase II—Mild Degeneration

The severity of disc degeneration is correlated with the levels of inflammatory mediators in the disc. As degeneration progresses, AF and NP cells and immune cells in the discs such as macrophages, neutrophils and T cells produce high levels of inflammatory cytokines including interleukin (IL)‐1*β*, IL‐6, IL‐17, IL‐2, IL‐8, IL‐10, IL‐4, tumor necrosis factor (TNF)‐*α*, interferon‐*γ* (IFN‐*γ*), and various chemokines such as C–C chemokine ligand 20 (CCL20), C–C chemokine receptor 6 (CCR6).^[^ ^12^, ^13^ ^]^ Proinflammatory cytokines such as IL‐1*β* and TNF‐*α* were found to regulate catabolic phenotype by upregulating matrix‐degrading enzymes, including MMP‐3, MMP‐13, and aggrecanases of a disintegrin and metalloprotease with thrombospondin motifs (ADAMTS)‐1, ADAMTS‐5, ADAMTS‐9 and 15 over ECM enzyme inhibitor of metalloproteinases (TIMP)‐1, TIMP‐2 and TIMP‐3 and downregulating the ECM expression of collagen, aggrecan, and SRY‐Box Transcription Factor (SOX)‐6.^[^ ^14^ ^]^ The TNF‐*α* and IL‐1*β* have also been reported to increase ADAMTS‐4, an important enzyme for aggrecan degradation via the MAPK and NF‐kB signaling pathways in the human NP.^[^ ^15^ ^]^ Additionally, TNF‐*α* and IL‐1*β* promote the expression of MMP‐3 through cooperative pathways of syndecan 4, MAPK and NF‐kB.^[^ ^16^ ^]^ Also, IL‐17 has been shown to mediate the inflammatory response via p38/*c‐Fos* and JNK/c‐Jun activation in an AP‐1‐dependent manner in the human NP.^[^ ^17^ ^]^

### 2.3 Phase III—Severe Degeneration

At a later stage, the disc loses its biomechanical function as a result of structural alterations. Fissures begin to form in the AF, causing the NP to extrude, allowing nerve‐ingrowth and vascularization in the disc, resulting in discogenic pain.^[^ ^18^ ^]^

Disc cells and the infiltrating immune cells of CD68+ macrophages, neutrophils and T cells (CD4+ and CD8+), continue to release cytokines and neurogenic factors including nerve growth factor (NGF) and brain‐derived neurotrophic factor (BDNF). The role of cytokines such as IL‐6 at the systemic level in disc disease has been demonstrated where back pain patients diagnosed with degenerative disc disease have higher serum cytokine levels. This suggests that such patients have low‐grade systemic inflammation.^[^ ^19^ ^]^ In a painful degenerated disc, IL‐1*β* and TNF‐*α* have been shown to upregulate brain‐derived neurotrophic factor (BDNF) and nerve growth factor (NGF) in the degenerated human disc, promoting innervation of small type of peptidergic nociceptive fibers into the aneural IVD, and vascularization induced by vascular endothelial growth factor (VEGF).^[^ ^20^ ^]^ These inflammatory changes together with sensitization of nociceptors, and pain processing neurons provide one explanation for the development of discogenic pain. However, in clinical practice these changes are seen almost universally in older patients many of whom are completely asymptomatic. Interpretation of scientific data on the structural changes in the disc in the context of clinical symptoms must be cautious as the relationship is not simply one of “cause and effect.”

Spinal stenosis, or narrowing of the spinal canal, often results in back pain as well as leg pain and the source of back pain can be difficult to determine. Degenerative disc disease results in loss of disc height which promotes proximal subluxation of the superior articular process of the facet joint, infolding of the ligamentum flavum and spinal stenosis. This leads to direct loss of lumbar lordosis which is then compounded by postural adjustments to rebalance the spine such as thoracic hyperlordosis and increased pelvic tilt. These compensatory mechanisms increase the workload of the paravertebral muscles leading to fatigue pain. This pain is typically worse on standing in one position and often relieved by rest. Structural changes in the degenerative disc including changes in vascularity and neural ingrowth may also result in backpain directly, independent of sagittal balance and muscle fatigue. Standing may also precipitate this pain as the disc is then loaded and relief follows from rest. In all likelihood, back pain associated with degenerative disc disease is multifactorial resulting from a combination of pain directly from the disc itself, neural compression and muscle fatigue due to poor sagittal balance.

While the specific pain generator may be difficult to identify in any given situation, preservation of disc integrity is likely to prevent the cascade of events which eventually leads to degenerative low back pain. Structural and biological changes in the disc architecture are probably the initiating events in this degenerative process leading to secondary changes in the facet joints and dimensions of the spinal canal. The advantages of regenerative therapies employing biomaterials and tissue engineering are the preservation of disc health and the initiation of this process to prevent or at least delay the onset of degenerative low back pain. This review is focused on these methods.

## 3 Current Clinical Treatments

Current treatments of LBP consist of a multimodal approach that includes pharmacological therapies, nonpharmacological options and surgical interventions.^[^ ^21^ ^]^

Pharmacological therapies have been specified in the guideline, with a single or combination of drugs. Medicines that are regularly prescribed include paracetamol, oral steroids, opioid analgesics, antidepressants, gabapentinoids, and muscle relaxants, in addition to nonsteroidal anti‐inflammatory drugs (NSAIDs).^[^ ^22^ ^]^ The high morbidity, mortality and addiction associated with opioids have piqued interest in using cannabinoids to treat LBP.^[^ ^23^ ^]^ Other pain‐relieving modalities, such as epidural injection, spinal manipulation and acupuncture used universally in clinical practice due to the lack of effective treatments with at least anecdotal benefits reported by patients.^[^ ^21^, ^24^ ^]^ Epidural injections are performed to treat chronic discogenic pain, administered via the lumbar epidural space by multiple routes including interlaminar, caudal, and transforaminal.^[^ ^6^ ^]^ A systematic review study indicated the effectiveness of epidural injections with local anesthetics and steroids for the treatment of discogenic low back pain with radiculitis.^[^ ^25^ ^]^ Hard evidence supports the use of injections of glucocorticoids or anesthetic agents into the epidural space, lumbar discs, lumbar facets, to treat chronic low back pain patients without radiculopathy.^[^ ^26^ ^]^

Physical therapy, exercise and manipulation resulted in some improvement, but little is known as to their effects over the long term.^[^ ^6^ ^]^ A Cochrane systematic review of randomized controlled trials revealed various exercises including strengthening, stretching exercises, and conventional physical therapy that consists of hot packs, massage, stretching, flexibility, and coordination exercises, proved more effective in reducing pain and improving functional disability, than was achieved by placebo.^[^ ^27^ ^]^ None of the less intensive rehabilitation programs, especially those not recommended by extensive components of behavioral therapy, or pain management programs relying on spinal injections and analgesic drugs, offer clear advantages over usual care for improving functional outcomes.^[^ ^28^ ^]^

The surgical management of degenerative disc disease is indicated once conservative management fails, presence of neurological deficit (i.e., cauda equina syndrome), spinal column deformity (i.e., degenerative kyphoscoliosis) or instability of functional spinal unit. Spinal surgery has conventionally worked best for spinal stenosis or radicular pain. The role of spinal surgery in spinal stenosis due to degenerative disc disease with symptoms of spinal claudication or radiculopathy is well established. Direct or indirect decompression for spinal stenosis (central or foraminal stenosis) with or without fusion procedures are common treatments in spine surgery. Surgical decompression as a stand‐alone technique has shown to be effective, especially with the introduction of minimally invasive procedures using numerous endoscopic systems.

Percutaneous minimally invasive procedures targeted to the intervertebral disc per se such as intradiscal steroids, and intradiscal thermal ablation using radiofrequency or laser devices have been advocated for the treatment of discogenic back pain. Annuloplasty is a procedure targeted to the annulus fibrosus (i.e., high intensity zone on MRI) by using electrothermal and radiofrequency ablation. The procedure can be adjunct to percutaneous lumbar discectomy. Procedures to alleviate discogenic pain such as intradiscal electrothermal therapy (IDET) modifies the collagen architecture of the disc making it thicker, and causing it to contract and decreasing its ability to revascularize.^[^ ^29^ ^]^ IDET also destroys (coagulates) the nociceptors within the annular region, thus inhibiting transmission of nociceptive input chemically or mechanically, thereby alleviating pain.^[^ ^30^ ^]^ Percutaneous disc decompression includes laser discectomy, radiofrequency ablation, mechanical disc decompression and manual percutaneous lumbar discectomy. The common laser (Clarus Medical) for the lumbar spine is holmium:yttrium‐aluminium‐garnet (Ho:YAG). The others are potassium‐titanyl‐phosphate and neodymium (Nd:YAG) laser. A percutaneous nucleotomy using cannulas aims to decrease intradiscal pressure to reduce irritation of the nerve root and the nociceptive receptors in the AF. Then, the use of cannulas reduces the risk of nerve injury, facilitates removal of the NP with an all‐in‐one suction cutting device, and decreases surgical time.^[^ ^29^ ^]^ Other devices such as Dekompressor (Stryker Corporation, Kalamazoo, MI, USA) were developed to remove NP tissue.^[^ ^29^ ^]^ However, the current evidence for these devices in treating low back pain is equivocal.

Spinal fusion is recommended when there is instability in the spine due to the pathology or after a decompression treatment.^[^ ^31^ ^]^ Spinal fusion techniques have evolved over the past century and various biologic agents have been developed to promote bony fusion.^[^ ^32^ ^]^ Clinical experience has demonstrated that mechanical back pain often persists despite bony fusion indicating that the disc is not necessarily the pain source. Far greater attention has recently been paid to the sagittal balance of the spine when planning surgical fusion as it has become apparent that the muscle is often the source of pain. Attaining sagittal balance by fusion has shown to be beneficial in degenerative spinal disorders.^[^ ^33^ ^]^ If careful attention is paid to the sagittal balance of the spine, then interbody fusion and elimination of the disc as a potential pain generator has been shown to significantly improve low back pain. A two‐year clinical trial revealed pain reduction and a greater functional improvement among patients who had spinal fusion surgery compared to patients who had unstructured physical therapy.^[^ ^34^ ^]^ Our clinical study demonstrated that spinal fusion has been shown to increase the quality of life in patients similar to healthy populations at similar ages, and patients underwent large joint replacement surgeries.^[^ ^35^ ^]^

## 4 Precision Medicine for Disc Tissue Engineering and Regenerative Therapy

Precision medicine is medical modeling of future advanced technology that explores the convergence of data sciences, analytic and medical research, including understanding the molecular basis of disease through multiomics profiling,^[^ ^36^, ^37^ ^]^ which aims to utilize patient information in developing an individualized treatment plan. In the case of IVD disease, precision medicine helps in designing targeted therapeutic strategies based on patient‐specific pathogenesis underlying IVD degeneration in the subgroup of the patient at early, mild to severe degeneration stages. The multifunctional smart biomaterial could be a key component of intervention contributing to advanced treatment for IVD regeneration.

**Figure 2:**
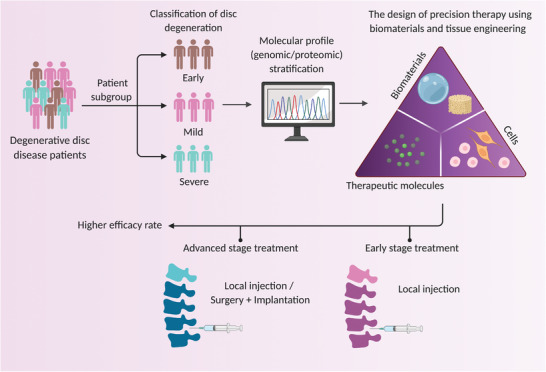
Precision medicine approach in IVD tissue engineering and regenerative therapy targeting early to severe IVD degeneration. Molecular profiling in subgroup of degenerative disc disease patients using genomic or proteomic approaches for patient stratification at early, mild and severe stages. Understanding patient‐specific pathogenesis IVD degeneration underlying discogenic low back pain will lead to identifying therapeutic targets and the design of biomaterials incorporating with targeted therapeutic molecules or cells tailored to disease severity. The decision of treatment plans through biomaterial injection alone at an early stage or the combination of biomaterials and therapeutic molecules or cells via the minimally invasive injection or surgical implantation at an advanced stage improves the treatment efficacy. The schematic was created with BioRender.com.

The combinatorial methods of precision medicine and biomaterial‐based tissue engineering would reduce the impact of patient heterogeneity, allowing for more precise patient stratification^[^ ^38^ ^]^ at early, mild and severe degeneration toward personalizing treatment plans.^[^ ^39^ ^]^ For example, the biomaterials can be designed alone in an injectable format to mimic and preserve the disc's extracellular matrix tailors to degenerative changes at the early stage of the disease. Therapeutic small molecules or drugs can be incorporated into biomaterials to halt the pathophysiology underlying mild disc degeneration. These biomaterials can be delivered through percutaneous minimally invasive procedures in addition to the current treatments at the early to mild stages of the disease. Biomaterials can also serve as an advanced cell delivery system to support the transplantation of cells such as stem cells to repopulate native cells in mild to severely degenerated discs associated with lower cellularity. The combination of biomaterials and cell therapy could be introduced via the injection through percutaneous minimally invasive procedures or surgical implantation (**Figure** **2**). Spinal fusion surgery has long been considered a last resort for the advanced treatment of lumbar degenerative discs. Despite the positive clinical outcomes of fusion surgery, adjacent segment disease or degeneration has been linked to long‐term complications of spinal fusion due to biomechanical changes in the spine. Here, the proposed biomaterial‐based tissue engineering therapy could delay the progression of disc degeneration in preventing repetitive spinal fusion in patients. Thus, combinatorial approach precision medicine and biomaterial therapy can prevent this complication by designing patient‐specific artificial disc and fusion devices. This can be done using additive manufacturing such as 3D printing to personalized biomaterials with the appropriate biomechanical and physicochemical properties tailoring anatomic features of the spine to maintain sagittal balance in patients, aiming to treat severely degenerated discs. Altogether, the proposed precision biomaterial‐based tissue engineering strategy tailoring severity of degenerative disc disease aims to provide a regenerative effect at early, mild and severe disc degeneration. This would enhance the treatment's efficacy, delaying the progression of the disease, preventing the surgical complications and improving quality of life in patients.

**Figure 3:**
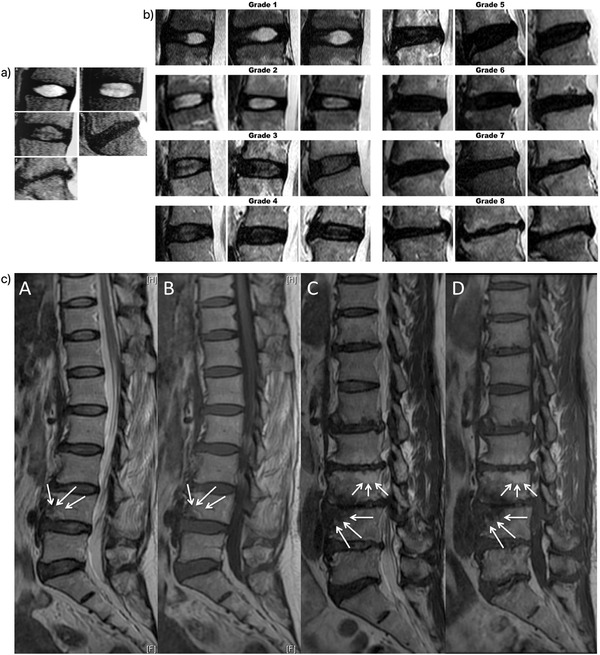
Classification system for IVD degeneration based on MRI pathological features. a) *Pfirrmann* grading system for the assessment of lumbar disc degeneration. Reproduced with permission.^[^ ^40^ ^]^ Copyright 2001, Lippincott Williams & Wilkins, Inc. A) Grade I: The structure of the disc is homogeneous, with a bright hyperintense white signal intensity and a normal disc height. B) Grade II: The structure of the disc is inhomogeneous, with a hyperintense white signal. The distinction between nucleus and annulus is clear, and the disc height is normal, with or without horizontal gray bands. C) Grade III: The structure of the disc is inhomogeneous, with intermediate gray signal intensity. The distinction between nucleus and annulus is unclear, and the disc height is normal or slightly decreased. D) Grade IV: The structure of the disc is inhomogeneous, with a hypointense dark gray signal intensity. The distinction between nucleus and annulus is lost, and the disc height is normal or moderately decreased. E) Grade V: The structure of the disc is homogeneous, with hypointense black signal intensity. The distinction between nucleus and annulus is lost, and the disc space is collapsed. Grading is performed on T2‐weighted midsagittal (repetition time 5000 msec/echo time 130 msec) fast spin‐echo images. b) Modified *Pfirrmann* grading system for lumbar disc degeneration. Image reference panel shows increasing severity of disc degeneration. Reproduced with permission.^[^ ^41^ ^]^ Copyright 2007, Lippincott Williams & Wilkins, Inc. c) Modic changes. Reproduced with permission.^[^ ^43^ ^]^ Modic type I change: hyperintense on T2WI (A arrow), hypointense on T1WI (B arrow) at inferior endplate of L4. Modic type II change: hyperintense on T2WI (C upper arrows), hyperintense on T1WI (D upper arrows) at superior endplate of L3. Modic type III change: hypointense on T2WI (C inferior arrows), hypointense on T1WI (D inferior arrow) at superior endplate of L4. Permission of figures adaptation from.^[^ ^40^, ^41^, ^43^ ^]^

Currently, the standard spinal magnetic resonance imaging (MRI) has been advocated for diagnosis and is used to classify IVD degeneration in patients. The *Pfirrmann* classification into five grades (grade I to grade V) adopted T2‐weighted MRI to indicate pathological changes in the disc, including structural homogeneity, the distinction of AF and NP, signal intensity, and disc height^[^ ^40^ ^]^ (**Figure** **3**a). The grade I is indicated by MRI homogeneous and bright white structure of the disc, clear distinction of the AF and NP; signal intensity is hyperintense and isointense to cerebrospinal fluid (CSF) and normal disc height. The grade II disc is identified by MRI inhomogeneous with or without horizontal bands of the disc, clear distinction of the AF and NP, signal intensity is hyperintense and isointense to CSF, and normal disc height. The grade III disc is identified with MRI inhomogeneous and gray structure of the disc, the unclear distinction between AF and NP, signal intensity is intermediate and normal to slightly decreased of disc height. The grade IV disc is indicated with MRI inhomogeneous and gray to the black structure of the disc, lost the distinction of the AF and NP, signal intensity is intermediate and normal to moderately decreased disc height. The grade IV disc is classified with the MRI inhomogeneous and black structure of the disc, the lost distinction between AF and NP, signal intensity is hypointense, and collapsed disc height.^[^ ^40^ ^]^ The *Pfirrmann* system was then modified by Griffith et al. for classification into eight grades (grade I to grade 8) of lumbar disc degeneration by including signal intensity from NP and inner AF, the distinction between inner and outer fibers of AF at the posterior aspect of the disc, and the disc height.^[^ ^41^ ^]^ Grade 1 is indicated by MRI signal intensity uniformly hyperintense and equal to CSF, distinct between inner and outer fibers of AF, and normal disc height. Grade 2 is classified when MRI is hyperintense (>presacral fat and <CSF) ± hypointense intranuclear cleft, distinct between inner and outer lamellae of AF, and disc height is normal. Grade 3 is identified as MRI hyperintense though <presacral fat, distinct between inner and outer fibers of AF, and normal disc height. Grade 4 is classified when MRI showed mildly hyperintense (slightly >outer AF fibers), indistinct between inner and outer AF fibers, and normal disc height. The MRI hypointense on NP and inner AF, and indistinct between inner and outer AF are the classification features for the grades 6 to 8, nevertheless, normal disc height is indicated for grade 5, <30% reduction of disc height for grade 6, 30–60% reduction of disc height for grade 7 and >60% reduction of disc height for grade 8^[^ ^41^ ^]^ (Figure 3b). However, the conventional T2‐weighted MRI and these classification systems are limited for detecting advanced disc degeneration, not at the early stage of degeneration. Therefore, the T1*ρ* relaxation time of MRI mapped on NP and AF was utilized in asymptomatic degenerative disc disease patients for quantitative measurement of biochemical changes correlated to interactions between macromolecules of ECM and water, which is associated with loss of ECM content such as proteoglycan, mainly to detect early IVD degeneration. A decreasing trend in T1*ρ* values has been indicated from the grade I to the grade IV of the *Pfirrmann* classification.^[^ ^42^ ^]^ Modic classification was also used for clinical assessment to describe the signal changes on MRI in the vertebral body adjacent to the endplates in addition to IVD degeneration.^[^ ^43^ ^]^ Modic changes on a vertebral endplate are significantly correlated with intervertebral disc degeneration. Type 0 is indicated by the normal signal of the endplate. Type I is identified by the endplate which shows regions that are hypointense on T1WI and hyperintense on T2WI. Type II is indicated by the endplate which shows regions that are hyperintense on T1WI and isointense or hyperintense on T2WI, but the signal changes are less marked than those for type I. Type III is identified by the endplate which shows hypointense regions on both T1WI and T2WI. The degeneration of the endplates is classified into four grades based on Modic changes: type 0 is grade 1, type 1 is grade 2, type 2 is grade 3, and type 3 is grade 4 (Figure 3c).

Besides, macroscopic morphology and histopathological scoring systems have also been established to assess the severity of IVD degeneration using *Thompson* classification, new histological classification and the recently standardized histopathology scoring system.^[^ ^44^, ^45^, ^46^ ^]^ However, these classification systems have more academic and research than clinical significance because they are based on assessing gross morphology and histopathological changes of the intervertebral disc and cannot be used on patients. Thompson utilized gross morphology of the NP, AF, endplate and vertebral body to classify the discs into five grades. Grade I is indicated by bulging gel of the NP, discrete fibrous AF lamellae, hyaline and uniformly thick endplate and margins of the vertebral body are rounded. Grade II is indicated by white fibrous tissue peripherally found in NP, mucinous materials between AF lamellae, the irregular thickness of endplate and rounded margins of the vertebral body are pointed. Grade III is indicated by consolidated fibrous tissue in NP, extensive mucinous infiltration in AF with loss of annular‐nuclear demarcation, focal defects in cartilage endplate and early chondrophyte or osteophytes at vertebral margins. Grade IV is indicated by NP horizontal clefts parallel to the endplate, focal disruption of AF, fibrocartilage extending from subchondral bone with irregularity, and focal sclerosis in the subchondral bone of the endplate and the vertebral body shows osteophytes less than 2 mm. Grade V is identified by a cleft extending through the NP and AF, diffuse sclerosis of the endplate and the vertebral body shows osteophytes greater than 2 mm.^[^ ^44^ ^]^

The advancement of the radiological approach in classifying disease severity with a biomarker or molecular profiling can improve diagnosis of degenerative disc disease. The establishment of disease classifications combined with molecular profiling can correlate the underlying pathology severity of IVD degeneration systemically and locally at early, mild, and severe stages. These biomarkers can be utilized as a prognosis tool, revealing valuable information about overall potential outcomes and providing information about the likelihood of treatment response. This information can aid in the optimization of final therapy selections.^[^ ^47^, ^48^ ^]^ The molecular profiling from degenerative discs at distinct severity levels of the disease will lead to the identification of key target markers associated with dysregulated signaling pathways underlying pathophysiology of disc degeneration. This can be useful for diagnostic measurement to be associated with patient's symptom (i.e., pain) and disease phenotype at early, mild and severe grades with the aid of MRI imaging. For example, target inflammatory mediators are key determinants of disc degeneration and pain mechanisms. The genome profiling of human discs has indicated upregulation of pain‐related markers, including bradykinin receptor B1, calcitonin gene‐related peptide (CGRP), catechol‐0‐methyltransferase, and nerve growth factor (NGF) in grades IV‐V degenerated discs in comparison to grade I‐III discs.^[^ ^49^ ^]^ Cytokine profiling revealed a higher serum level of IL‐6 was found in LBP patients with lumbar disc disease.^[^ ^19^ ^]^ A recent study reported a correlation of whole blood C‐C chemokine receptor 6 and IL‐6 gene expression levels in patients diagnosed with lumbar disc disease.^[^ ^13^ ^]^ Robust increasing of local inflammatory cytokines and catabolic enzymes (MMPs and ADAMTS) has been shown to mediate IVD degeneration.^[^ ^11^ ^]^ Our recent study has revealed an altered glycosylation in grade V human degenerative disc, which was characterized by increased expression of sialylated and fucosylated N‐glycosylation motifs, might play a role in inflammation and disease progression.^[^ ^50^ ^]^ Hydration is another important hallmark of IVD degeneration. A spatiotemporal proteome profiling of the human discs revealed the depletion of matrisome proteins associated with loss of tissue hydration in the aged discs.^[^ ^51^ ^]^ The phenotypic study of notochordal and progenitor markers of the IVDs is also essential to elucidate the microenvironment and heterogeneous population in AF and NP cells.^[^ ^52^ ^]^

Overall, identifying target markers from profiling study of the IVD degeneration at early, mild and severe stages will give an idea of designing and developing the next generation of patient‐specific biomaterial‐based tissue engineering therapy, which can be tailored to the severity of the IVD degeneration. For example, biomaterials can be designed to mimic the microenvironment of the NP or AF based on the study of ECM profiling in healthy NP and AF tissue. The information of ECM profiling (i.e., molecules, proteins and glycans composition) is valuable for selecting type of biomaterials to be developed toward an optimal and conducive microenvironment of the IVD. The optimal biomaterials are also essential in supporting transplantation of cells (stem cells, AF/NP‐like cells or notochordal‐like cells) to preserve AF and NP phenotype for long‐term efficacy. Biomaterials can also be functionalized in more personalized manner with small molecule or inhibitor or drug to tailor the key determinants underlying pathogenesis early, mild and severe disc degeneration. Finally, research into identification of target markers from profiling study of healthy and subgroup of IVD degeneration at early, mild, and severe stages are necessary to improve understanding of IVD homeostasis and patient‐specific pathophysiology of the disease, which lead to the development of patient‐specific biomaterial‐based tissue engineering therapy.

## 5 Rationale Precision Biomaterial‐Based Tissue Engineering Targeting Severity of the Intervertebral Disc Degeneration

The future direction of the biomaterial approach in tissue engineering lies in designing biomaterial‐based tissue engineering tailored to the severity stages of the IVD degeneration to enhance the efficacy of the treatment. The clinical success of therapy is determined by the relief of pain and disability in patients. Herein, precision biomaterial‐based tissue engineering aims to address the pathogenesis underlying IVD degeneration at early, mild and severe stages of the disease. This information will be used for the development of patient‐specific biomaterials and tissue engineering strategy to halt the disease progression, promote tissue regeneration and relieve the symptom, i.e., discogenic low back pain. Excellent biocompatibility, biodegradability, sufficient biomechanical properties, and a chemical or structural architecture that allows the diffusion of metabolites, nutrients, and regulatory molecules inside and outside cells, are all key requirements for the selecting for IVD tissue engineering. the capacity of the biomaterials to respond to the host‐environment, maintain disc phenotype, attain tissue biochemical homeostasis and promote ECM deposition for anatomical tissue repair, thus providing functional mechanical support.^[^ ^53^ ^]^ **Table** **1** summarizes the rationale of personalized biomaterial‐based tissue engineering for IVD regeneration, targeting early to mild and mild to severe IVD degeneration.

**Table 1:** Precision biomaterial‐based tissue engineering for disc regeneration targeting severity of the disease

Scaffold	Cell source for testing/model	Descriptions	Analysis	Ref.
Early intervertebral disc degeneration				
*Creating IVD‐specific matrices to maintain cellular phenotype*				
A. Xenograft biomaterials				
Porcine NC‐rich NP matrix (NCM)	In vitro adolescent and adult NP cells from bovine	ECM anabolism	GAG content.	^[^ ^54^ ^]^
Porcine NC‐rich NP matrix (NCM)	In vitro HUVECs	Antiangiogenesis	HUVEC vessel formation (length measurements).	^[^ ^55^ ^]^
B. ECM mimetics				
Natural‐inspired biomaterials				
Type I collagen microsphere	Rabbit NP cells encapsulated in microsphere3D in vitro	Microstructure growth kinetics Cell distribution Cell morphology Cell viability ECM anabolism	Diameter of microsphere using scanning electron microscopy (SEM). Cell density by Trypan blue staining. NP rounded cells by phase‐contrast light microscope. Live/dead staining. Glycosaminoglycan (GAG) content by Alcian blue staining. Type II and I collagen by collagen antibody staining. Sulfated GAG by dimethylmethylene blue (DMMB) assay.	^[^ ^60^ ^]^
Rivoflavin‐crosslinked collagen gel	In vitro rat caudal intervertebral discs motion segments	Biomechanical integrity ECM anabolism	Collagen remained in place and increased the effective equilibrium modulus following stress‐relaxation testing in steps of compressive strain. Proteoglycan by Safranin‐O and fast green staining.	^[^ ^57^ ^]^
Thiolated HA (HA‐SH) and PEG vinylsulfone (PEG‐VS) hydrogels	Porcine AF and NP cells	Biomechanical integrity ECM anabolism Metabolite Morphology	Hydrogel property ranged 70–489 kPa. Higher sGAG production by DMMB assay. Secretion of lactate and pyruvate concentration. Multicell clusters visualized by light microscopy.	^[^ ^58^ ^]^
Type II collagen hydrogel crosslinked star‐PEG, enriched with HA	Rabbit adipose‐derived stem cells (ADSCs), and encapsulation of bovine NP cells in hydrogel	Functionalization Enzymatic stability Gelation; mechanical Cell viability Cell proliferation Cell distribution ECM anabolism	Primary amine groups of collagen by 2,4,6‐trinitrobenzene sulfonic acid assay (TNBSA). Degradation by collagenase assay. Gelation time and storage modulus by rheology. ADSC viability by Trypan blue. NP cell viability by live/dead assay. NP cell proliferation by PicoGreen assay. NP distribution by stereology. Type I/II collagen and aggrecan by qPCR.	^[^ ^61^ ^]^
Chitosan (CHI) solutions filled with cellulose nanofibers	In vitro human dermal fibroblast cultured on hydrogels. Intradiscal injection of hydrogel in ex vivo porcine and rabbit spine model	Biomechanical integrity Ultrastructure Cell viability Hydration Microstructure Injectability Biomechanical integrity	Viscosity, storage modulus, and loss modulus under frequency sweep test by rheology. Scaffold morphology by Atomic force microscopy (AFM) and SEM. Viability of fibroblast by live/dead assay. Cytotoxicity determined by metabolic activity using 3‐[4,5‐dimethylthiazol‐2‐yl]‐2,5‐diphenyltetrazolium bromide (MTT) assay. Hydration indicated by magnetic. resonance imaging (MRI). Disc stained for hematoxylin and eosin (H&E). Intradiscal injection of hydrogel using 25G needle for in situ gelation without leakage. Fiber mechanical reinforcement under the compressive loads.	^[^ ^62^ ^]^
Alginate–collagen porous composite	AF and MSCs of porcine 4 months‐old	Shape memory Microstructure Cell morphology ECM anabolism Mechanical integrity Cell migration Cell viability Cell proliferation ECM anabolism Cell viability	Swelling ratio. SEM for porosity. H&E for cell colonization. Picro‐Sirius Red for collagen deposition. Compressive equilibrium modulus. Release profile TGF‐*β*3. In vitro AF cell migration: Cell cytoskeleton fluorescence staining, cell morphology using SEM, DNA content. Cyto‐compatibility of MSCs. Fibroblastic‐like cell morphology. Cell proliferation. Deposition of collagen type I, TGF‐*β*3. Ex vivo bovine organ culture of defect model: maintained MSC viability.	^[^ ^63^ ^]^
Ionic‐(iGG–MA) and photo‐crosslinked (phGG–MA) methacrylated gellan gum hydrogels	MSCs, nasal chondrocyte, Human dermal microvascular endothelial cells	MSCs phenotype Cell proliferation Cytotoxicity Inflammation ECM anabolism Apoptosis Microstructural degeneration	Surface marker CD29, CD44, CD73, CD90, CD105, Alizarin red for tri‐lineage differentiation, osteogenic differentiation by Von Kossa, adipogenicity by Oil red O, chondrogenicity by Safranin O and Alcian blue staining. Proliferation by crystal violet and 3‐(4,5‐dimethylthiazol‐2‐yl)‐5‐(3‐carboxymethoxyphenyl)‐2‐(4‐sulfophenyl)‐2H‐tetrazolium (MTS) assays. ELISA for IL‐8 and ICAM‐1. Immunohistochemistry (IHC) for collagen II, aggrecan and vimentin. Cell death by Terminal deoxynucleotidyl transferase dUTP nick end labeling (TUNEL) assay. TEM for subcutaneous implantation in severe combined immunodeficient (SCID) mice by IHC.	^[^ ^64^ ^]^
Porous silk fibroin (SF) scaffolds	In vitro rabbit NP cells; subcutaneous implantation in nude mice model	Ultrastructure Cell viability Cell proliferation Cell distribution ECM Anabolism Biomechanical integrity Cell viability ECM anabolism	High porosity, pore size and high pore interconnectivity by optical microscopy, SEM. Viability by Trypan blue and live/dead assay. DNA content by Hoechst dye. Stained NP cell–scaffold by H&E staining. Proteoglycan and type II collagen by ELISA. Compressive elastic modulus by compression speed. Tracing of implanted cell‐scaffold using in vivo live imaging (IVIS) and 4,6‐diamino‐2‐phenyl indole (DAPI). Proteoglycan and collagen by H&E staining.	^[^ ^67^ ^]^
Hyaluronan‐poly(*N*‐isopropylacrylamide) (HA‐pNIPAM)	Human MSCs	Cell viability DNA content ECM anabolism Disc phenotype Cell viability ECM anabolism	Live/dead cytotoxicity Quant‐IT PicoGreen assay DMMB assay for sulfated GAG. Type I/II collagen, aggrecan, cytokeratin‐19, SOX9, fork‐head box protein F1, cluster of differentiation 24, and carbonic anhydrase 12 by qPCR. Bovine caudal ex vivo: DNA content and live/dead assay. GAG content, and gene expression.	^[^ ^68^ ^]^
Synthetic biomaterials				
Laminin‐111 functionalized PEG hydrogel	Porcine notochordal cells	Functionalization Cell adhesion ERK pathway Mechanical property Cell survival	Free amine group by TNBSA. Cell viability on PEG‐Laminin‐111 coated on well plates using CellTiterGlo. Level of phosphorylated ERK by ELISA. Storage (*G*′) and loss (*G*″) moduli; gelation time by rheology. Cell viability post‐injection of Luciferase expressing porcine NP cells in the nucleotomy ex vivo motion segments of rat IVD and rat tail model	^[^ ^70^ ^]^
Copolymer hydrogel of laponite, pNIPAM and *N*′‐dimethylacrylamide (DMAc)	human adult mesenchymal stem cells (hMSCs)	Physical property Mechanical property Viscosity Swelling Hydration Gelation Cell viability Microstructure ECM anabolism NP phenotypic markers	Analysis of dynamic light scattering (DLS) for the formation of a liquid hydrogel. Analysis of dynamic mechanical on solidified hydrogel. Analysis of viscosity by glass capillary Ostwald viscometer. Swelling capacity. Hydration degree by wet weight. Gelation time. Metabolic activity by AlamarBlue assay. Interior surface morphology by SEM. ECM deposition by H&E, Alcian blue, Alizarin Red, Masson Trichrome staining. Expression of aggrecan, chondroitin sulfate, type II collagen, paired box 1 (PAX1), hypoxia‐inducible factor, forkhead box F1 (FOXF1) by IHC.	^[^ ^69^ ^]^

*Cell–ECM interaction for the regulation of cellular functions, cytoskeletal organization and extracellular matrix biosynthesis*				
Type II collagen microgel crosslinked star‐PEG, supplemented with HA	3D in vitro rabbit ADSCs encapsulated in microgel	Cell viability Cell proliferation Cell morphology Stress fibers Glycogen content NP phenotype	ADSCs viability by live/dead assay. ADSCs proliferation by flow cytometry. Live cell by Calcein staining. Cell's cytoskeleton with phalloidin staining. Glycogen in cell's cytoplasm by TEM. High levels of type II collagen, sex‐determining region Y‐box 9 (SOX‐9), aggrecan, and low levels of type I collagen by qPCR.	^[^ ^75^ ^]^
Disc‐like angle‐ply structures (DAPS)	Implantation of acellular DAPS in rat's caudal discs	Stability Mechanical integrity Cell infiltration ECM anabolism Anatomical restoration	Gross macroscopic revealed intact AF region, while the NP has contracted. Compressive moduli, maximum strain. Cell infiltration. Tissue deposition by Alcian blue, Picosirius staining. Types I and II collagen by IHC. Adjacent vertebral bone remodeling by micro‐computed tomography (CT).	^[^ ^53^ ^]^

Mild intervertebral disc degeneration				
*Therapeutic molecules to regulate cellular responses for tissue repair*				
Gelatin microparticles coloaded with Matrilin3/TGF‐*β*3	Seeding microparticle with ADSCs to form spheroid in vitro, intradiscal injection of spheroid in the rat tail model	Morphology Degradation Kinetic release Chondrogenesis Hypertrophy Anabolism Hydration	Round morphology, solid, smooth surface and diameter size of microparticles indicated by SEM. Slow enzymatic degradation in collagenase I. Slow release of TGF‐*β*3 over 15 days. Spheroid formation marker of Ki‐67 in ADSCs. Chondrogenic markers of SOX9, aggrecan and collagen II by RT‐qPCR. Hypertrophy markers of COLX, MMP13, and RUNX2 by RT‐qPCR. Expression of collagen II in spheroid and in the disc by immunohistochemistry, and TGF‐*β*1, TGF‐*β*2, and TGF‐*β*3 by RT‐qPCR in vitro, proteoglycan stained by Masson Trichrome. MRI indicated for disc hydration.	^[^ ^88^ ^]^
Polymer capsules of (poly (allylamine hydrochloride), dextran, tannic acid loaded with catalase	In vitro inflammation of NP cells	Morphology Surface charge Physicochemical Enzyme incorporation Oxidative scavenging capacity Viability Capsule uptake Oxidative stress Catabolism Inflammation	Spherical shape indicated by SEM, and hollow of capsules imaged by TEM. Zeta‐potential for capsules using DLS. Removal of CaCO_3_ template indicated by FTIR and EDX. Stop flow measurement. Bradford assay. Fluorimetric assay to measure hydrogen peroxide, and electron paramagnetic resonance measurements for hydroxyl radical. Metabolic activity by AlamarBlue and live/dead. FITC‐labeled dextran, cytoskeletal staining by rhodamine Phalloidin. CellRox staining for oxidative stress. Expression of MMP‐3 and ADAMTS‐RT‐qPCR, and NF‐k*β* by western blot. IL‐6 level determined by ELISA.	^[^ ^89^ ^]^

*Cell factories to promote endogenous disc regeneration*				
Collagen II/HA/CS tri‐copolymer	Implantable of allograft rabbit NP seeded scaffold in nucleotomy rabbit model	Cell morphology ECM anabolism Anatomical restoration Disc height Disc hydration	Viable allografted cells with chondrocyte‐like shape by H&E staining. Prominent staining of Alcian blue for ECM. Increase disc height index by plain radiograph. Restore T2 signal intensity on MRI.	^[^ ^94^ ^]^
Soluble or bound pentosan polysulfate (PPS) in PEG/HA hydrogel	Encapsulation of human mesenchymal precursor cells on hydrogel	Functionalization Mechanical integrity Swelling property Hydrolytic stability Cell proliferation Cell viability ECM anabolism Mechanical integrity Cell viability Cell distribution ECM anabolism	Amine‐functionalized‐HA measure by TNBSA. Carboxylation of PPS and amine‐functionalized‐ha by FTIR, nuclear magnetic resonance spectroscopy (NMR). Gelation kinetics and final moduli by rheology. Degree of swelling in PBS. Degradation in PBS. MPC proliferation by cell counting kit 8 (CCK8) and EdU staining proliferation kit (EdU) assay. MPC viability by live/dead assay. Increase GAG synthesis by DMMB. Alcian blue, toluidine blue and safranin O staining for ECM content. Rheology: Time sweep analyses for elastic shear modulus, Frequency sweeps test for the elasticity of materials. Viability of encapsulated cells in hydrogel by live/dead. MPCs distribution in hydrogel by H&E. Turnover of the pericellular matrix, deposition of GAGs in hydrogel by safranin O, Alcian blue, and Picrosirius red staining. Increase type I/II collagen by IHC.	^[^ ^95^ ^]^
Tyramine‐functionalized HA/PEG (3‐4‐hydroxyphenylpropionic acid) crosslinked H_2_O_2_ hydrogel	Stro3‐selected human MPCs encapsulated in the hydrogel in vitro; acellular hydrogel implantation in rat subcutaneous in vivo	HA functionalization Mechanical integrity Swelling property Hydrolytic stability Injectable system Cell viability Cell distribution	Amine‐functionalized‐HA by NMR. Gelation kinetics and final moduli by rheology. High degree of swelling in PBS. Degradation test in PBS. In vitro injection in hydrated agarose gel. MPC viability by live/dead assay. Cell distribution in hydrogel by H&E.	^[^ ^96^ ^]^

*Attenuation of inflammation*				
Type II collagen/HA and collagen I hydrogel	Bimodular bovine NP and AF cells in HA/collagen hydrogel of disc inflammation model	Cell morphology, and distribution Cell viability Cell proliferation Neuroinflammation Genotyping Glycosylation	F‐actin staining for cell shape and distribution. Cell viability by live/dead assay. PicoGreen for cell proliferation. qPCR for type I/II collagen, aggrecan. NGF and BDNF mRNA expression by qPCR. ELISA for IL‐6 and TNF‐*α* levels. Genomic profile by RNA sequencing. Lectin histochemistry of SNA‐I (Sambucus nigra agglutinin I), MAA (Maackia amurensis agglutinin), WGA (Wheat germ agglutinin), Con A (Concanavalin A), UEA‐I (Ulex europaeus agglutinin I), PNA(Peanut agglutinin), GS‐IB4 (Griffonia simplicifolia isolectin), WFA (Wisteria floribunda agglutinin), LEA (Lycopersicon esculentum agglutinin).	^[^ ^97^ ^]^
HA/PEG hydrogel	Normal and inflamed NP cells seeded on hydrogel in vitro	Functionalization Stability Degradation Cell viability Inflammation Cell surface Neuroinflammation	Un‐crosslinked of HA (carboxyl groups) by FTIR, and amount of amine groups (PEG) by TNBSA Incubation in PBS. Incubation in hyaluronidase. NP viability live/dead. NP metabolic activity by AlamarBlue assay. IHC for IL‐1R1, MyD88. IHC for CD44. qPCR for NGF and BDNF.	^[^ ^98^ ^]^

Severe intervertebral disc degeneration				
*Alleviation of pain*				
HA/PEG hydrogel	Implantation of acellular hydrogel in disc pain injury model of rat tail	Mechanical allodynia Thermal hyperalgesia Nociception Hyperinnervation Inflammation ECM anabolism Glycosylation	Pain behavior assessment by Von Frey test. Pain behavior assessment by Hargraves test. qPCR for c‐Fos and substance P. IHC for GAP43, CGRP, TrkA, TRPV1. ELISA for proinflammatory cytokines IL‐1, IL‐6. IHC for SMAD family member 3 (SMAD3), COLI, aggrecan, fibronectin. Lectin staining for SNA‐I, MAA, GS‐I‐B4, WFA, WGA, keratan and chondroitin sulfate.	^[^ ^99^ ^]^

*Support disc's mechanical properties*				
Injectable self‐assembled peptides: GAG hydrogel	Injection of hydrogel into denucleated bovine IVDs ex vivo	Mechanical integrity	Gelation, stability, effusion of injected material, compressive stiffness in denucleated bovine IVDs.	^[^ ^106^ ^]^
In situ oxidized HA/gelatin hydrogel	Rabbit NP cells seeded on hydrogel; NP cells encapsulated in the hydrogel in vitro	Functionalization Mechanical integrity Cell viability Cell morphology NP phenotype	Confirm oxidized HA/gelatin by Fourier‐transform infrared spectroscopy (FTIR). Storage moduli (elasticity) and loss modulus (viscosity) under oscillation frequency sweep tests by rheology. NP viability by live/dead and Alexa Fluor 488 and Rhodamine Phalloidin. Cell‐seeded hydrogel morphology by SEM. qPCR for HIF‐1, SOX‐9, biglycan (BGN), decorin (DCN), aggrecan (ACAN), COL1A1 and COL2A1.	^[^ ^107^ ^]^
Thiolated HA/poly(ethylene glycol)vinylsulfone hydrogel	Porcine AF and NP cells seeded on top of the hydrogel	Mechanical integrity ECM anabolism DNA content Metabolite Cell morphology, and distribution	Torsional dynamic shear for storage modulus (*G*′) and complex modulus by rheology. Sulfated GAG compositions. DNA stained with Hoechst dye. Lactate and pyruvate concentration by colorimetric reaction kit. Cell clustering and spreading by light microscope.	^[^ ^108^ ^]^

*Replicate anatomic features of the IVD*				
Riboflavin crosslinked high‐density collagen gel	Annular defects needle‐punctured rat‐tail model	Stability Anatomical restoration Hydration Mechanical integrity	Distribution, integration, and reorganization of postinjection of FITC‐labeled collagen gel. Safranin O and Alcian blue for tissue reorganization and retention of NP. MRI *Pfirrmann* grading. Stress‐relaxation tests; hydraulic permeability.	^[^ ^110^ ^]^
Hierarchical anisotropic nanofibrous laminates (electrospinning poly(*ε*‐caprolactone) (PCL))	MSCs seeded between bilayer tissue construct in vitro	ECM anabolism Mechanical integrity	Deposition of sulfated GAG and collagen; a thin interlamellar space by Alcian blue and Picosirius red. Increased uniaxial tensile moduli during in vivo compression of the IVD.	^[^ ^111^ ^]^
Disc‐like angle‐ply structures (DAPS) (electrospinning of aligned PCL and poly(ethylene oxide) (PEO) layers)	Bovine AF cells in vitro; implantation of DAPS in rat caudal *discs* in vivo	Disc height Stability ECM anabolism Cell infiltration Anatomical restoration	Disc height by lateral fluoroscopy imaging. Matrix deposition and lamellar structure by H&E. Cell infiltration by DAPI staining. Adjacent vertebral bone remodeling by micro‐CT scan.	^[^ ^112^ ^]^
3D Silk Fibroin/elastin bioprinted scaffold	Cultured human ADSCs on the scaffold	Physicochemical properties Stability Hydration Mechanical property Microstructure Proliferation Viability	Chemical composition and structural conformation by FTIR. Weight loss ratio for enzymatic degradation in protease XIV. Water uptake ratio in phosphate buffer saline (PBS). Elastic modulus under compressive test. Viscoelastic measurements by dynamic mechanical analysis Surface morphology by SEM. Ultrastructure by high‐resolution micro‐CT. DNA content by PicoGreen. Metabolic activity by Alamar Blue, and viability by Live/dead assay.	^[^ ^113^ ^]^

### 5.1 Early Intervertebral Disc Degeneration

The early stage of IVD degeneration is mediated by an imbalance of ECM biosynthesis that leads to cellular physiology changes toward senescent phenotype, low cellularity, NP phenotype changes and inflammation in the IVD. Designing a biomaterial by creating IVD specific matrices using ECM‐based biomaterials in mimicking microenvironment of the IVD to maintain cellular phenotype, and promote cell–ECM interaction in regulating cellular functions, cytoskeletal organization and ECM biosynthesis in the early IVD degeneration, thus preventing mild degeneration.

#### 5.1.1 Creating IVD‐Specific Matrices to Maintain Cellular Phenotype

The ECM‐based biomaterials, including xenograft biomaterials^[^ ^54^, ^55^ ^]^ and ECM mimetics, are the most promising strategy to develop IVD matrices due to their excellent cytocompatibility, low immunogenicity, and tunable mechanical properties.^[^ ^56^ ^]^ Biomaterials that are natural, semisynthetic, or synthetic are commonly employed to create ECM mimetics. Natural biomaterials such as collagen,^[^ ^57^ ^]^ hyaluronic acid (HA),^[^ ^58^ ^]^ and among others have been developed with tunable macromolecule compositions and concentrations to provide instructive biochemical cues toward a desired cellular phenotype. The ECM macromolecules provide physical scaffolds to mimic the IVD niche and regulate cellular functions, including growth, migration, survival, and homeostasis, thereby maintaining cellular phenotype.^[^ ^59^ ^]^ Because of the avascular and low cell density nature of the IVD, ECM‐based biomaterials in hydrogel form become the most favorable platform to create 3D scaffolds while retaining their biological properties. For example, microencapsulation of NP cells in the 3D microspheres system of collagen I demonstrated a round morphology of NP cells that maintained NP phenotypic markers of type II collagen and cytokeratin‐19.^[^ ^60^ ^]^ A collagen II hydrogel has increased cell viability without affecting the NP phenotype.^[^ ^61^ ^]^ Other natural biomaterials are chitosan,^[^ ^62^ ^]^ gelatin,^[^ ^63^ ^]^ alginate,^[^ ^63^ ^]^ gellan gum,^[^ ^64^ ^]^ fibrin,^[^ ^65^, ^66^ ^]^ and silk fibroin^[^ ^67^ ^]^ that has been used to create IVD matrices. Semisynthetic biomaterial such as poly(N‐isopropylacrylamide (pNIPAM) grafted with HA to form a thermoresponsive hydrogel that has been found to support human mesenchymal stem cells (hMSC) differentiated toward IVD phenotype, including collagen type II (COLII), SOX9, cytokeratin‐19, CD24, and forkhead box protein (FOXF1).^[^ ^68^ ^]^ Semisynthetic biomaterials such as fibrin and poly(lactic‐*co*‐glycolic acid) (PLGA) scaffold has provided a 3D microenvironment for AF and NP cells to develop cells cluster morphology, maintain cellular phenotype of type II collagen and aggrecan, with a higher cell proliferation and proteoglycan‐rich matrix and glycosaminoglycan (GAG) content over 14 days in culture.^[^ ^66^ ^]^ In contrast, synthetic biomaterials adopt a “bottom‐up” approach to mimic natural ECM.^[^ ^69^ ^]^ For example, laminin‐111 functionalized PEG hydrogel has demonstrated higher cell survival postinjection in the IVD explant model.^[^ ^70^ ^]^

#### 5.1.2 Cell–ECM Interaction for the Regulation of Cellular Functions, Cytoskeletal Organization, and Extracellular Matrix Biosynthesis

The ECM‐based biomaterials provide a conducive microenvironment for cells to regulate theirs functions include viability, proliferation, adhesion, and the synthesis of ECM proteins and other macromolecules, which influence multiple signaling pathways and provide protection from the hostile local injection or implantation environments.^[^ ^53^ ^]^ The cell–ECM interaction has evidently dictated cell morphology, survival, differentiation, proliferation, ECM biosynthesis through cell surface receptor‐mediated signaling.^[^ ^71^ ^]^ For example, the HA acts as a signaling molecule to bind with cell surface receptors, including CD44, the receptor for hyaluronan mediated motility (RHAMM), and the HA‐binding PG versican to regulate cytoskeletal organization, cell adhesion, and cell migration.^[^ ^72^ ^]^ Collagen is another example of an ECM macromolecule that regulates the expression of integrin receptors via the outside‐in signaling associated with cytoskeleton organization.^[^ ^73^ ^]^ It also play a role in cell adhesion and migration via interactions with glycoproteins in the extracellular matrix, resulting in cellular differentiation.^[^ ^74^ ^]^ We previously reported that collagen II microgels supplemented with HA induced differentiation of adipose‐derived stem cells (ADSCs) toward NP phenotype, expressing markers of aggrecan, collagen II, and SOX9, while suppressing stress fibers formation and marker of cell length (ROCK 1), possibly through crosstalk between integrin *α*10 and CD44 interactions.^[^ ^75^ ^]^ Laminins are ECM glycoproteins comprise of *β*, *α*, and *γ* chains that regulate cell survival, adhesion, differentiation, and migration. A study reported that a series of laminin‐mimetic peptide poly(ethylene) glycol (PEG) hydrogels modulated integrin and syndecan binding in NP cells, thus promoting ECM biosynthesis^[^ ^76^ ^]^ primarily through integrin *α*3 mediated interaction with laminin, which also associated with phosphorylation of signaling molecules of extracellular‐signal‐regulated kinase (ERK1/2) and glycogen synthase kinase (GSK3*β*).^[^ ^77^ ^]^

### 5.2 Mild Intervertebral Disc Degeneration

The IVD degeneration progresses with prolonged depletion of ECM synthesis, lower cellularity, increased catabolic phenotype, which modulate multiple cellular signaling toward the inflammatory and degenerative niche. Biomaterials can be designed as an advanced delivery system incorporating therapeutic molecules to regulate cellular signaling, modulating cellular responses, including anticatabolic, antioxidative, anti‐inflammatory, and anabolic effects for tissue repair. To address the lower cellularity nature of IVD degeneration, biomaterials can be combined with cells to recruit endogenous tissue regeneration. Therapeutic biomaterials can be introduced to target inflammatory signaling‐mediated IVD degeneration, in which inflammation is the primary indication of IVD degeneration.

#### 5.2.1 Therapeutic Molecules to Regulate Cellular Responses for Tissue Repair

Recent advancement of biomaterials as a delivery system has provided a broad range of benefits in tissue engineering and regenerative medicine.^[^ ^78^ ^]^ Biomaterials can transport exogenous therapeutic molecules, including growth factors (PDGF, FGF, IGF‐I, and TGF‐*β*1),^[^ ^79^, ^80^, ^81^, ^82^ ^]^ cellular regulator, drug or inhibitor (anti‐TNF*α*, IL‐1ra, celecoxib, rapamycin, NF‐*κ*B decoy)^[^ ^83^, ^84^, ^85^, ^86^ ^]^ from the cargo to the target site specifically to regulate cellular signaling, thereby dictating cellular responses, including anticatabolic, antioxidative, antiapoptotic, anti‐inflammatory, and anabolic effects for IVD regeneration.^[^ ^87^ ^]^ This delivery system provides sustained release of the therapeutic molecules over time for a long‐term efficacy. For example, gelatin microparticles coloaded with TGF‐*β*3 and ECM protein matrilin 3 enabled slow release of these factors over 15 days, which had shown upregulation of chondrogenic markers in adipose‐derived mesenchymal stem cells (ADSCs) for anabolic effects (SOX9, aggrecan, collagen II) in vitro and promoted tissue repair on ECM deposition of collagen II and hydration in the IVD at 8 week in vivo study.^[^ ^88^ ^]^ Inflammation has been associated with increased reactive oxygen species (ROS). Antioxidative catalase loaded polymer capsules functionalized with tannic acid prevented oxidative stress in cells and downregulated catabolic phenotype (MMP‐3, ADAMTS‐5) in an in vitro NP inflammation model.^[^ ^89^ ^]^ Bai et al. reported that in situ‐formed ROS‐scavenging scaffold loaded with rapamycin (Rapa@Gel) has been shown to increase expression of anti‐inflammatory M2‐like macrophage phenotype polarization over M1‐like macrophages, decrease local inflammatory microenvironment, and increase disc height and MRI intensity in degenerative caudal disc model, indicating tissue regeneration in vivo.^[^ ^90^ ^]^ Recently, we reported that a small molecule of sialyltransferase inhibitor (3Fax‐Peracetyl Neu5Ac;2F‐Peracetyl‐Fucose) was capable of modulating cell migration and catabolic enzymes in inflamed human NP cells over seven days in culture, which could be a therapeutic target to inhibit sialylated *N*‐glycosylation that mediate inflammation in IVD degeneration.^[^ ^50^ ^]^

#### 5.2.2 Cell Factories to Promote Endogenous Disc Regeneration

Cell‐based therapy aims to repopulate the cellularity of IVD and promote tissue remodeling through ECM biosynthesis by implanted cells, thereby influences native cell's function. The cells can be delivered alone or through an injection or implantation within a biodegradable scaffold. Biomaterials scaffold act as a cell carrier by providing physical support for cell placement, promoting specific cellular microenvironments, and secreting efficacious factors that target cellular function for regeneration.^[^ ^91^ ^]^ Transplantation of autologous NP or AF cell; exogenous cells including allogenic, xenogenic NP or AF cells, multipotent stem cells, predifferentiated mesenchymal stem cells (MSCs) in vitro; and endogenous cell stimulation on notochordal cells and MSCs using growth factors or proteins were widely adopted to replicate IVD homeostasis for intrinsic tissue regeneration.^[^ ^92^, ^93^ ^]^ For example, seeding the NP cells on a tri‐copolymer construct of collagen II/HA/chondroitin‐6‐sulfate has been shown to promote ECM deposition and increase the disc height index in a rabbit model of nucleotomy.^[^ ^94^ ^]^ Mesenchymal progenitor cells (MPCs) seeded on PEG/HA‐based hydrogels incorporated with pentosan polysulfate (PPS) has demonstrated chondrogenic differentiation, higher cell viability, exhibited a rounded morphology of cells and increased ECM deposition.^[^ ^95^ ^]^ Furthermore, an MPC encapsulated hydrogel revealed good cell viability and promoted cartilage‐like tissue with rounded morphology, increased collagen II depositions in vitro, and did not exhibit any macroscopic signs of an inflammatory response in a rat subcutaneous implantation model.^[^ ^96^ ^]^

#### 5.2.3 Attenuation of Inflammation

An increase in inflammation has been associated with painful discs. Targeting local inflammatory response would be a promising strategy to prevent pain. Our previous study reported that a bioengineered collagen II and HA hydrogel revealed inflammatory crosstalk on a molecular basis of ECM production, neurotrophins, and suppressor of cytokine signaling in the inflamed IVD providing an appropriate disease model in vitro to study disc inflammation.^[^ ^97^ ^]^ In an inflammation model of NP cells, we reported a therapeutic biomaterial of a PEG crosslinked‐HA‐based hydrogel that has been demonstrated to reduce IL‐1*β* signaling molecules and neurogenic markers, including NGF and BDNF, potentially through the interaction of CD44.^[^ ^98^ ^]^ We also observed a decrease of IL‐6 and IL‐1*β* levels in the AF and NP tissues postimplantation of HA hydrogel in the injured discs of the rat tail model. A similar trend of systemic inflammatory markers was decreased in HA hydrogel treatment.^[^ ^99^ ^]^ The HA hydrogel has also shown to attenuate interferon (IFN‐*α*) signaling molecules, interferon‐induced protein with tetratricopeptide repeats 3 (IFIT3) and proapoptotic insulin‐like growth factor‐binding protein‐3 (IGFBP3) in the bovine IVD explant model.^[^ ^100^ ^]^ Another example, a free radical scavenger, fullerol nanoparticles has been shown to reduce proinflammatory mediators of IL‐6, IL‐1*β*, PGE2, and cyclooxygenase‐2 in TNF‐*α* stimulation of an in vitro model of mouse.^[^ ^101^ ^]^

### 5.3 Severe Intervertebral Disc Degeneration

The IVD structure becomes disorganized, inhomogeneous, and collapses lead to alteration of IVD biomechanical function. An increase of neuroinflammation‐mediated IVD degeneration has been implicated with an increase of severity of IVD degeneration. Neuroinflammation is defined as the presence of a neuroinflammatory process in the tissue.^[^ ^102^ ^]^ In the case of IVD, it has been linked with an increase of proinflammatory cytokines and neurogenic factors to induce expression of pain‐related markers (i.e., ion channels and neuropeptides) in the IVD and dorsal horn of spinal cord that contribute to the development of low back pain.^[^ ^12^, ^101^ ^]^ In particular, a long‐term increase of proinflammatory cytokines such as IL‐1*β* has been demonstrated to stimulate neurotrophins such as NGF and BDNF to promote hyperinnervation of nociceptive nerve fibers and peripheral sensitization, which initiate nociception.^[^ ^20^ ^]^

#### 5.3.1 Alleviation of Pain

**Figure 4:**
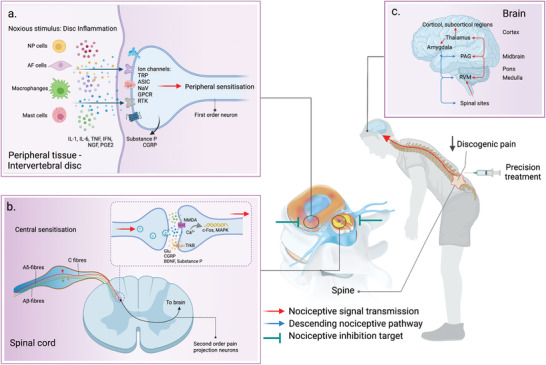
Schematic inflammatory pain processing and inhibitory target at peripheral and spinal levels through local injection IVD treatment to produce an antinociceptive effect. a) At peripheral tissue, disc degeneration causes the release of inflammatory and neurogenic mediators (IL‐1, IL‐6, TNF, IFN, NGF, and PGE2) by resident cells, including AF and NP cells, macrophages, and mast cells. The inflammatory signaling molecules activate nociceptors by binding to cell surface receptors or ion channels such as voltage‐gated sodium channels, transient receptor potential cation channel (TRP) channels, receptor tyrosine kinases (RTK), G protein coupled receptors, acid‐sensitive ion channels (ASIC) on first‐order neurons, thus initiating membrane depolarization for transmitting action potentials to the spinal cord. b) Action potentials are transmitted along nociceptive fibers in the DRG to the presynaptic neurons in the dorsal horn. Prolonged noxious stimulation activates C and A*δ* fibers to release excitatory neurotransmitters (glutamate, substance P, CGRP, and BDNF) onto synapses in lamina I of the dorsal horn to mediate central sensitization through NMDA glutamate receptors or TrkB receptor. This neurotransmission can increase intracellular calcium to activate calcium‐dependent signaling pathways and second messengers (c‐Fos, MAPK, and protein kinase), increasing the excitability of the postsynaptic neurons to transmit pain to the brain. c) The axons of second‐order neurons decussate at the spinal cord and anterolaterally project to the thalamic nuclei and brainstem. The third‐order neurons project to several cortical regions encodes for sensory‐discriminative aspects of pain. Overall, the inhibition of nociceptive transduction at peripheral tissue will suppress persistent noxious stimulation at the spinal level to inhibit excitatory glutamatergic and peptidergic neurotransmissions up to supra‐spinal level, thereby producing an antinociceptive effect. The schematic was created with BioRender.com.

The next generation of therapeutic biomaterials and functionalization of biomaterials with analgesic agent can be formulated to target neuroinflammatory responses and nociception in the discs, in particular, biomaterials can inhibit hyperinnervation of nociceptive fibers in the NP and AF. These nociceptive nerve fibers contain neuropeptides originating from the dorsal root ganglion (DRG), small diameter NGF‐sensitive neurons,^[^ ^103^ ^]^ which play a critical role in inflammation‐induced hyperalgesia. Thus, attenuation of neuroinflammation will inhibit hyperinnervation of nociceptive nerve fibers, suppressing peripheral sensitization of ion channels such as voltage‐gated sodium channels, transient receptor potential cation channel (TRP) channels, receptor tyrosine kinases (RTK), G protein coupled receptors, acid‐sensitive ion channels (ASIC), resulting in blocking the action potential for nociceptive transduction and transmission in spinal cord level up to the brain to exhibit the analgesic effect (**Figure** **4**). In the study of disc pain, a HA hydrogel reduced postoperative fibrosis and radicular pain in a rat model of postlaminectomy.^[^ ^104^ ^]^ In our recent study, we revealed PEG crosslinked‐HA based hydrogel to prevent inflammatory pain specifically to alleviate mechanical allodynia and thermal hyperalgesia in the disc injury model of rats through downregulation of hyperinnervation (GAP43, CGRP and TrkA) and nociception (TRPV1, c‐Fos, and substance P).^[^ ^99^ ^]^ We also found that injecting PEG‐crosslinked HA‐based hydrogel into the punctured discs at the lumbar discs reduced inflammation, and sensory innervation (PGP9.5), thus halting the progression of IVD degeneration in a rabbit model.^[^ ^105^ ^]^

#### 5.3.2 Support Disc's Mechanical Properties

Fine tuning of the macromolecular composition of biomaterials for viscoelasticity, injectability or implantability, and physical or functional stability are key elements that affect the biomechanical properties of the formed network support the disc's function mechanically. A hydrogel scaffold becomes the most promising results in IVD repair since the native NP tissue is composed of a highly hydrated ECM. The unique physicochemical property of glycosaminoglycan (GAG), particularly HA, to absorb a large amount of water molecules has made it a favored choice of material for restoring disc hydration. For example, an injectable glutamine‐ and serine‐based peptides self‐assembling in situ hydrogels enriched with GAG chondroitin sulfate exhibited higher gelation kinetics and thermodynamic stability to improve stiffness following compressive loading in denucleated IVD bovine ex vivo model.^[^ ^106^ ^]^ A HA/gelatin hydrogel exhibited higher viscoelastic properties (11–14 kPa) under a complex shear modulus as similar to native NP behavior (11.3 kPa).^[^ ^107^ ^]^ An enzymatically crosslinked PEG/HA hydrogel displayed a higher storage modulus ranging from 5.55 to 5.46 kPa and exhibited predominantly elastic behavior over three months.^[^ ^108^ ^]^ Posttreatment of electrospun polycaprolactone (PCL) scaffold and fibrin–genipin (FibGen) adhesive hydrogel exhibited higher minimum strain than the intact disc in an endplate delamination injury model for AF repair.^[^ ^109^ ^]^

#### 5.3.3 Replicate Anatomic Features of the Intervertebral Disc

The successful strategy to engineer AF or NP replacement relies on the biomaterial selection and their assembling into the anatomic architecture of the load‐bearing IVD.^[^ ^110^ ^]^ For example, the concentric ring of AF has a unique angle‐ply microstructure that provides higher mechanical property in the IVD. Mainly, an aligned nanofibrous scaffold was fabricated as a single lamellar by electrospinning poly(*ε*‐caprolactone) (PCL) and seeded with MSCs over 2 weeks in culture before being coupled into bilayers in an orientation either parallel (+30°/+30°) or opposing (+30°/−30°). The scaffold has demonstrated to promote sulfated GAG and collagen deposition that replicated the direction of angle‐ply and multilamellae of AF, thus providing higher uniaxial tensile moduli.^[^ ^111^ ^]^ In combination with the external fixation system, the nanofibrous disc‐like angle‐ply (DAPS) was tested in the rat tail model that increased disc height and histologically demonstrated minimal superficial adjacent vertebral bone deposition and lamellar structure.^[^ ^112^ ^]^ Another personalized approach for IVD tissue engineering is 3D printing, an emerging rapid prototyping technology precisely tuning the architecture of tissue‐engineered scaffold using biomaterials for disc replacement. For example, silk fibroin hydrogel combined with elastin have been used for 3D printing to develop the patient‐specific AF implants in mimicking the anatomic features and mechanical properties of native AF, which demonstrated higher cell viability and proliferation in vitro.^[^ ^113^ ^]^

## 6 Ongoing and Concluded Therapeutic Clinical Trials

In recent years, there has been an increased interest in the paradigm shift from spinal surgeries toward biological therapies that promise to restore disc anatomy and function. These emerging concepts are underpinned by the potential of bioengineered therapies to induce multitissue regeneration processes even at mechanically challenging sites such as the spinal column. Many of the scientific analyses performed over the past two decades have identified key signaling pathways as well as candidate biomolecules and pluripotent cells that could be of therapeutic interest. Nevertheless, questions remain as to how many preclinical studies reported are reproducible in patients. Indeed, given the plethora of research work focusing on biological therapies to restore the disc anatomy and mechanical properties, only a handful of bioengineered treatments are currently undergoing clinical trials, as revealed by a search of the websites www.ClinicalTrials.gov. Of the fifteen clinical trials reported so far (**Table** **2**), eight studies involve the implantation of cells.

**Table 2:** Clinical trials in degenerative disc disease

Clinical trial ID	Biological agent (s)	Group	Eligibility (years old)	Enrolment (patients)	Country	Study period	Remarks
NCT02338271	Autologous adipose derived mesenchymal stem cells (ADSCs) with hyaluronic acid derivatives (Tissuefill)	Experimental ADCS (2 × 10^7^ cells mL^−1^ per vial or 4 × 10^7^ cells mL^–1^ per vial) plus Tissuefill (hyaluronic acid derivatives) 1 mL per syringe	18–70	10	Korea	January 2015 to December 2017	Status—unknown Inclusion criteria—LBP, who have failed to respond to conservative treatments ≥3 months, degenerative disc *Pfirrmann* grade III–IV, VAS score ≥40 mm, ODI ≥30%. Exclusion criteria—subjects with severe lumbar stenosis, herniated NP, spinal instability, spondylitis, or vertebral fractures, severe IVD degeneration with disc height collapse ≥50%, severe osteoporosis, patients have received intradiscal procedures, lumbar epidural steroid injection, hypersensitive to sodium hyaluronate, pregnancy or breastfeeding, severe medical disease.
NCT02097862	Autologous adipose derived stem cells	Experimental Intradiscal injection of adipose derived stem cells	18–85	100	USA	March 2014 to January 2017	Status—completed. Pain relief with no reported adverse effects Inclusion criteria—subjects of degenerative disease with LBP after conservative treatment ≥6 months, fibrous ring capable of holding the cell implantation, absence of spinal infection. Exclusion criteria—congenital spine deformation, spinal instability, spinal canal stenosis, isthmus pathology, Modic III changes, reduction ≥50% of disc height, pregnant or nursing females, life expectancy <6 months active infectious disease, chronic immunosuppressive treatment.
NCT01643681	Autologous adipose tissue derived stem cells	Experimental Autologous adipose tissue derived mesenchymal stem cell transplantation	19–70	8	Korea	July 2012 to December 2015	Status—completed. Awaiting publication of results Inclusion criteria—lumbar intervertebral disc degeneration, degenerated intervertebral disc on T2‐weighted MRI, chronic LBP > 1 year, failed ≥1 year of nonoperative low back pain management, positive discography, lumbar instability at degenerated disc. Exclusion criteria—lumbar herniated intervertebral disc, pregnant and nursing females, alcohol or drug abuse, preexisting medical conditions.
NCT01640457	NOVOCART autologous disc chondrocyte transplantation	Experimental NOVOCART Disc plus (autologous disc chondrocyte transplantation System) Comparator NOVOCART Disc basic (media with no active cell component) Sequestrectomy (standard of care)	18–60	120	Austria Germany	August 2012 to June 2021	Status—active. No adverse effects observed so far. Inclusion criteria—subjects with disc herniation with back and/or leg pain, single‐level lumbar disc herniation, remaining ≥50% disc height, no signs of osteophytes, patient has an indication for sequestrectomy, without adjacent degenerative disc, patients with adjacent degenerative disc with *Pfirrmann* 3–4, but no more than 25% disc height reduction. Exclusion criteria—patients had a previous surgery at the lumbar level(s) and a past recurrent disc herniation treated with sequestrectomy, apparent degenerative changes in the lumbar spine as determined by Modic Changes 2–3, dysplastic vertebral bodies within the lumbar spine, sacralized lumbar vertebra LWK5 at the level to be treated, spondylodiscitis, Segmental instability (spondylolisthesis >5 mm) or translation >3 mm 6, isthmic spondylolisthesis, ankylosing spondylitis or spondylolysis, lumbar scoliosis (>11° deformation, previous trauma, discography or any other surgical intervention at the lumbar spine, other conditions—any degenerative muscular or neurological condition, BMI >35 kg m^−2^, history of illicit drug, nicotine (more than 20 cigarettes per day) or alcohol abuse, CRP >10 mg dL^−1^, pregnant, breastfeeding, history of known allergies, Immune defects/suppression, patient has active systemic or local microbial infection, eczematization or inflammable skin alterations at the site of surgery, chronically inflammable character (i.e., arthritis, gout), osteoporosis, hyperparathyroidism or hyperthyroidism, Systemic connective tissue, ocular degenerations, blood coagulation disease, had undergone chemo or radiotherapy.
NCT01771471	Allogenic juvenile chondrocytes in fibrin NuQu	Experimental Single administration of NuQu treatment in fibrin carrier Comparator Single administration of saline	21 and older	44	USA	November 2012 to September 2020	Status—over 50% of patients continued to improve at 1 year follow‐up. Inclusion criteria—LBP aggravated by movement and or postural changes ≥6 months, failed conservative management, *Pfirrmann* Grade III or IV degenerated lumbar disc. Exclusion criteria—disc extrusion at any level in their lumbar spine; disc bulges or protrusions at any level in the lumbar spine resulting in nerve root compression, more than 50% loss of disc height, more than 50% loss of disc height, Type I Modic changes at any level other than the targeted level, Type I Modic changes at the treated level, Osteoporotic compression fracture, Lumbar Scheurmann's disease, Antero or retrolisthesis ≥3 mm at any level, experiencing chronic pain generating from any other source, infection at the treatment site, immune‐deficiency or immunosuppressive, BMI ≥ 40, comorbid conditions, history of alcoholism, medication or intravenous drug abuse, contraindications for MRI.
NCT01860417	Allogenic mesenchymal stromal cells	Experimental MSCs prepared from bone marrow from healthy donor expanded ex vivo for 3–4 weeks Intradiscal injection of 25 million MSC in 2 mL of saline Active comparator Infiltration of paravertebral musculature close to the affected disc(s) with 2 mL of 1% Mepivacaine	18–75	25	Spain	April 2013 to April 2017	Status—completed. Awaiting publication of results Inclusion criteria—degenerative disease of one or two lumbar discs with predominant back pain, conservative treatment (physical and medical) ≥6 months, fibrous ring capable of holding the cell implantation, reduction of disc height <20%, absence of spinal infection, no alterations on hematological and biochemical analysis. Exclusion criteria—spinal segmental instability, spinal canal stenosis, isthmus pathology, Modic III changes on MRI images, allergy to gentamicin, bovine, cattle or horse serum, congenital or acquired diseases, BMI >35, pregnancy or breast‐feeding, neoplasia, immunosuppression.
NCT01290367	Allogeneic mesenchymal precursor cells + hyaluronic acid	Experimental Injection of high dose MPCs with hyaluronic acid Injection of low dose MPCs with hyaluronic acid Comparator Intradiscal injection of saline solution. Intradiscal injection of hyaluronic acid solution	18 and older	100	USA	August 2011 to July 2015	Status—completed. Awaiting publication of results Inclusion criteria—chronic LBP ≥6 months, diagnosis of DDD of one level from L1‐S1, change in disc hydration on MRI with or without an annular fissure or a contained disc herniation, failed 3 months of nonoperative LBP management, disc height loss of <30%, at least 40 mm on a 100 mm VAS, LBP greater than leg pain, ODI ≥ 30. Exclusion criteria—stenosis or frankly herniated disc or sequestered discs, Intact disc bulge/protrusion or focal herniation at the symptomatic level (s) >3 mm or presence of disc extrusion or sequestration, lumbar spondylitis or other undifferentiated spondyloarthropathy, had undergone a previous surgery at the involved levels, previous lumbar intradiscal injection procedure, fracture of the spine, history of epidural steroid injections, hypersensitivity to murine or bovine products and hyaluronan, have been a recipient of prior stem cell/progenitor cell therapy, pregnancy or breast‐feeding, history within the last 3 years of neoplasm.
NCT02412735	Allogeneic mesenchymal precursor cells Rexlemestrocel‐L + hyaluronic acid	Experimental Rexlemestrocel‐L 2.0 mL injection of approximately 6 million rexlemestrocel‐L cells in freeze media mixed in a 1:1 by volume ratio with saline Rexlemestrocel‐L 2.0 mL injection of approximately 6 million rexlemestrocel‐L cells in freeze media mixed in a 1:1 by volume ratio with hyaluronic acid Comparator Saline solution	18 and older	404	USA, Australia	March 2015 to June 2021	Status—completed Inclusion criteria—degeneration of an intervertebral disc from L1 to S1, *Pfirrmann* score of 3, 4, 5, or 6, Modic Grade II changes or less, with or without contained disc protrusion at the index disc, chronic LBP >6 months at least ≥40 mm and not more than 90 mm of VAS, leg pain ≤20 mm of VAS in both legs, ODI ≥30 and no more than 90, failed 6 months of conservative LBP management, have at a minimum undergone supervised physical therapy and taken a pain medication for back pain (i.e., NSAIDs and/or opioid medication. Exclusion criteria—*Pfirrmann* score of 7 or 8 at any lumbar level, symptomatic involvement of more than one lumbar disc and central vertebral canal stenosis as defined by neurogenic claudication, spondylolysis, spondylolisthesis or retrolisthesis grade 2, Lumbar spondylitis, spinal deformity, any fracture of the spine, facet pain, full thickness annular tears, patients have undergone a surgical procedure (i.e., discectomy, intradiscal electrothermal therapy, intradiscal radiofrequency, artificial disc replacement, interbody fusion), undergone intradiscal injection procedure of (contrast medium, NSAIDs, nerve‐blocking anesthetics, antibiotics, saline), undergone procedures affecting spine structure/biomechanics (posterolateral fusion), spinal litigation, sacroiliac joint pain, stenosis or disc protrusion, malignancy or tumor, have been a recipient of prior allogeneic stem cell/progenitor cell therapy, baseline morphine equivalent dose (MED) of >75 mg per day, underlying medical conditions, alcohol or drug abuse, pregnancy or breast‐feeding, BMI ≥ 40, osteoporosis.
NCT01158924	Recombinant human growth and differentiation factor‐5 (rhGDF‐5)	Experimental Intradiscal injections of rhGDF‐5	18 and older	40	Australia	March 2010 to March 2014	Status—completed. Awaiting publication of results. Inclusion criteria—persistent LBP ≥ 3 months of nonsurgical therapy at one or two suspected symptomatic lumbar levels (L3/L4 to L5/S1), LBP with ODI of ≥30 and VAS of ≥4 cm. Exclusion criteria—chronic radiculopathy, active radicular pain due to anatomical compression such as stenosis or disc herniation, symptomatic facet joints, symptomatic sacroiliac joint.
NCT01182337	Recombinant human growth and differentiation factor‐5 (rhGDF‐5)	Experimental Intradiscal injection of rhGDF‐5 Comparator Vehicle control of trehalose, glycine, HCl, and water for injection	18 and older	31	Korea	June 2010 to June 2014	Status—completed. Awaiting publication of results. Inclusion criteria—persistent LBP > 3 months of nonsurgical therapy at one or two suspected symptomatic lumbar levels (L3/L4 to L5/S1), LBP with ODI of ≥30 and VAS of ≥4 cm. Exclusion criteria—chronic radiculopathy, active radicular pain due to anatomical compression (i.e., stenosis or disc herniation) symptomatic facet joints, extravasation of contrast agent during the discogram.
NCT00813813	Recombinant human growth and differentiation factor‐5 (rhGDF‐5)	Experimental Intradiscal injections of rhGDF‐5	18 and older	32	USA	June 2008 to June 2013	Status—completed. Awaiting publication of results Inclusion criteria—persistent LBP ≥ 3 months of nonsurgical therapy at one or two suspected symptomatic lumbar levels (L3/L4 to L5/S1), LBP with ODI of ≥30 and VAS of ≥4 cm. Exclusion criteria—unable to have discogram, chronic radiculopathy, active radicular pain, leak of contrast agent during the discogram, decrease in disc height >50%, Modic changes, and/or presence of osteophytes or significant facet arthrosis.
NCT01124006	Recombinant human growth and differentiation factor‐5 (rhGDF‐5)	Experimental Intradiscal injection of rhGDF‐5 Comparator Vehicle control of sterile water for injection	18 and older	24	USA	January 2010 to September 2014	Status—completed. Awaiting publication of results Inclusion criteria—persistent LBP ≥ 3 months of nonsurgical therapy at one suspected symptomatic lumbar levels (L3/L4 to L5/S1) confirmed by provocative discography, LBP with ODI ≥30 and VAS of ≥4 cm. Exclusion criteria—unable to have discogram, chronic radiculopathy, active radicular pain, extravasation of contrast agent during the discogram, symptomatic facet joints and/or severe facet joint degeneration.
NCT03197415	Autologous growth factor of platelet‐rich plasma gel	Experimental Intradiscal injection of platelet‐rich plasma gel	20 – 60	30	Taiwan	April 2016 to March 2019	Status—completed. Awaiting publication of results Inclusion criteria—lumbar spine disc degeneration, LBP. Exclusion criteria—herniated disc, history of spine surgery, cancer, and hematological disease, pregnancy.
NCT02320019	Drug of YH1461	Experimental Intradiscal injection of YH1461 A mg/disc Intradiscal injection of YH1461 B mg/disc Intradiscal injection of YH1461 C mg/disc Intradiscal injection of YH1461 D mg/disc Comparator Placebo matching YH14618	19 and older	326	Korea	March 2015 to August 2016	Status—completed. Awaiting publication of results Inclusion criteria—patients diagnosed as one or two symptomatic early lumbar (L1/L2 to L5/S1) degenerative disc disease with *Pfirrmann* grade 2–4, persistent LBP ≥3 months conservative therapy, LBP of VAS ≥ 4 cm and ODI ≥ 30%. Exclusion criteria—spine compression fracture, spinal stenosis, or spinal instability, sacroiliac joint dysfunction, facet joint pain, Modic change type III, history of spine surgery, neurologic disorders, systemic disease or participated in other clinical trials with intradiscal injection.
NCT01694134	Drug of corticoids	Experimental Intradiscal Injection of hydrocortancyl Comparator Intradiscal injection of lidocaine	18 – 80	50	France	July 2012 to March 2017	Status—completed. Awaiting publication of results Inclusion criteria—LBP ≥6 months, Modic I discopathy. Exclusion criteria—lumbar surgery, patient under anticoagulant or antisludge treatment, unbalanced diabetes mellitus (blood glucose >1.30 g L^−1^), unstabilized high blood pressure (>160/95 mmHg), infection, iodine allergy or one of the Hydrocortancyl or Lidocaine components, cauda equine syndrome, untreated psychotic state, pregnancy, vulnerable persons.

In contrast, four studies involved the intradiscal application of a growth factor of recombinant human growth/differentiation factor 5 (rhGDF‐5), and one study used platelet‐rich plasma to induce disc regeneration. One study involved intradiscal injection of 7‐amino acid peptide in inhibiting TGF*β*1‐induced NGF expression, and one study has finished trials for intradiscal injection of corticoids. Of the eight studies of cell implantation, three have focused on the potential of adipose tissue‐derived autologous stem/stromal cells with HA derivative (NCT02338271) and without biomaterials (NCT01643681, NCT02097862). Two studies are testing the use of either autologous disc chondrocytes (NCT01640457) or allogenic juvenile cultured chondrocytes (NCT01771471) with fibrin, respectively, to promote disc tissue repair. Three trials evaluate allogeneic mesenchymal precursor cells with the carrier of HA (NCT01290367, NCT02412735) and mesenchymal stromal cells without a carrier (NCT01860417).

## 7 Clinical Perspective and Future Directions

Current experimental models of biomaterials and tissue engineering therapy have revealed efficacy to maintain disc phenotype, regulate cellular functions (viability, proliferation, differentiation, and cell adhesion) and extracellular matrix organization and secretome through multiple signaling pathways, promote tissue regeneration and therapeutic responses (antioxidative and anticatabolic), attenuation of inflammation and nociception as well as mechanical support, especially in animal studies. However, evidence of therapeutic success is still limited in human trials. The primary outcomes of biomaterials and tissue engineering in clinical studies should focus on the treatment‐emergent adverse events, improvement of disc height index, disc volumetry and signal intensity, improvement of visual analogue scale for LBP and quality of life evolution in patients. Other parameters are vital signs, physical examination and clinical laboratory analysis, including key biomarkers for disc phenotype, inflammation and pain.

The main challenge in the clinical fields is that decision making is mainly guided by the patient history of pain and disability with clinical signs in addition to radiological imaging, including MRI. This idea leads to the concept of inflammation that contributes to disc‐related back pain. Current strategies aim to develop the technology by focusing on biological targets (including inflammation), laying the groundwork for better diagnosis. Besides improving MRI imaging analysis, it may also necessitate biomarker testing to understand biochemical cues underlying the structural and functional state of the degenerative disc. Personalized biomaterial‐based tissue engineering approaches are tailored to regenerate the degenerated disc with the prospect of enhancing the future management of LBP with more a favorable outcome compared to the current therapeutic armamentarium. Furthermore, personalized treatments will be possible with a precise diagnosis. It is time to translate the basic science of biologic‐mechanistic IVD into effective therapeutics capable of alleviating the discogenic LBP in patients suffering from IVD degeneration.^[^ ^114^ ^]^

The immediate future, therefore, lies in the ability of the scientific community to achieve one or all of the following four: 1) to prepare a “fertile ground” using biomaterials for the survival of remaining disc cell populations to improve functionality in situ; and 2) to fine‐tune the functions of biomaterials to recruit specific cell populations that would produce therapeutic factors at efficacious doses to rescue degenerated disc tissues; 3) to elucidate long‐term efficacy of biomaterials and its mechanism of actions to promote disc regeneration and alleviate inflammation and pain; 4) to personalize biomaterial‐based tissue engineering tailor to the severity of the IVD degeneration targeting discogenic LBP.

## 8 Conclusions

The comprehensive understanding of the pathology of IVD degeneration from cellular and tissue levels, biochemical and ECM alterations, and molecular pathways underlying early, mild, and advanced disease stages will offer future opportunities to develop efficacious therapeutics that are essential for developing clinically appropriate strategies for IVD degeneration targeting discogenic LBP. Biomaterial‐based tissue engineering strategies have demonstrated efficacy in regulating local tissue responses, maintaining disc phenotype, attaining tissue biochemical homeostasis, and promoting anatomical tissue repair in preclinical models. However, despite intensive research efforts focused on biological therapies to prevent the advanced stages of IVD degeneration, only a handful of bioengineered therapies are currently undergoing open clinical trials. The lack of clinical evidence to date to support these therapies to date is likely to reflect the relatively short term follow up in a process that takes decades to evolve. It may well be that the secondary effects of degenerative disc disease such as spinal stenosis and muscle fatigue produced by loss of lumbar lordosis and sagittal balance of the spine are responsible for more pain than the structural changes in the disc themselves. If this is the case, then the clinical effect of advances to prevent disc degeneration will only be evident in studies designed to assess the development of these secondary effects. Invariable this will require long‐term follow‐up.

## Conflict of Interest

The authors declare no conflict of interest.

## Author Contributions

I.L.M.I. and A.P. assisted in article conception. I.L.M.I., S.A.M., S.A.A., M.B.F., and A.D. assisted in literature survey and writing. I.L.M.I. assisted in editing. A.P., S.A.M., and A.D. assisted in reviewing. All the authors provided the final approval.
